# Outdoor Wood Mats-Based Engineering Composite: Influence of Process Parameters on Decay Resistance against Wood-Degrading Fungi *Trametes* *versicolor* and *Gloeophyllum* *trabeum*

**DOI:** 10.3390/polym13183173

**Published:** 2021-09-18

**Authors:** Minzhen Bao, Neng Li, Yongjie Bao, Jingpeng Li, Hao Zhong, Yuhe Chen, Yanglun Yu

**Affiliations:** 1 Key Laboratory of High Efficient Processing of Bamboo of Zhejiang Province, China National Bamboo Research Center, Hangzhou 310012, China; lineng8657@sina.com (N.L.); bomithen@126.com (Y.B.); lijp@caf.ac.cn (J.L.); zhonghao@caf.ac.cn (H.Z.); yuhec@sina.com (Y.C.); 2 Research Institute of Wood Industry, Chinese Academy of Forestry, Beijing 100091, China

**Keywords:** outdoor wood mats-based engineering composite, wood-degrading fungi, decay resistance, durability

## Abstract

The process parameters significantly influence the preparation and final properties of outdoor wood mats-based engineering composite (OWMEC). During outdoor use, wood composites are susceptible to destruction by rot fungi. Herein, the role of process parameters such as density and resin content on OWMEC resistance to fungal decay was investigated. The poplar OWMEC samples were exposed to white-rot fungus *Trametes versicolor* and brown-rot fungus *Gloeophyllum trabeum* for a period of 12 weeks. The chemical composition, crystallinity, and morphology were evaluated to investigate the effect of process parameters on the chemical composition and microstructure of the decayed OWMEC. With an increase in the density and resin content, the mass loss of the decayed OWMEC decreased. The highest antifungal effect against *T. versicolor* (12.34% mass loss) and *G. trabeum* (19.43% mass loss) were observed at a density of 1.15 g/m^3^ and resin content of 13%. As results of the chemical composition and microstructure measurements, the resistance of OWMEC against *T. versicolor* and *G. trabeum* fungi was improved remarkably by increasing the density and resin content. The results of this study will provide a technical basis to improve the decay resistance of OWMEC in outdoor environments.

## 1. Introduction

Wood, as a renewable material, is in high demand for construction and building applications. It can be converted into engineered wood composites with standardized dimensions, such as wood oriented strand board, glue laminated wood, and reconstituted wood lumber. Scrimber, a promising type of engineered wood composite, is marketed as being moisture resistant and suitable for outdoor structural use [1]. Wood mats-based engineering composite (WMEC), as a novel engineered scrimber composite, consists of mechanically defibered wood fiber mats soaked with a phenolic resin and compacted to up to three times the original density of the wood using hot or cold pressing [2]. The development of WMECs refers to the defibration technology, bonding technology, and forming process [3,4].

During outdoor use, wood and its composites are susceptible to discoloration by mold fungi, destruction by rot fungi, and attack by insects. Biological attack limits the utilization of wood materials because of physical, chemical, and biological changes occurring on the surface and inside the material. Discoloration by mold fungi can cause high-value loss of wood composites when exposed to humid or moist conditions [5]. Biodeterioration by white and brown rot fungi can alter the chemical composition of wood [6]. Witomski et al. [7] reported that the bending strength and compressive strength of Scots pine wood can decrease by up to 20% with only 7% mass loss. The outdoor service life of wood composites is closely related to their deterioration under ambient conditions.

The degradation rate of wood composites is related to internal parameters such as the moisture, density, and resin content. Kataoka et al. [8] reported that the spreading rate and extent of fungal degradation were dependent on the density of the composite. Furthermore, accelerated weathering is more likely to occur in low-density composites [9]. Previous studies have indicated that the dimensional stability and photostabilization of wood composites can be improved by increasing the concentration of phenol formaldehyde (PF) resin [10,11]. To improve the durability of wood composites, the common industrial practice is to adjust the resin content to over 13% and density up to 1.10 g/cm³. The mass loss of bamboo scrimber after 12 weeks of fungal erosion was less than 5% [12,13]. However, the decay resistance of WMECs has not been adequately investigated, especially for different densities and resin contents.

In order to expand the applicability and extend service lifetime of outdoor WEMC (OWMEC), more attention should be paid to its decay resistance. Impregnation of PF resin and compression of wood have been shown to improve the durability and dimensional stability of wood [14,15]. Hence, the objective of this study was to investigate the resistance of OWMECs with different densities and resin contents exposed to *Trametes versicolor* and *Gloeophyllum trabeum* fungi for a period of 12 weeks. The wet chemical method, Fourier transform infrared spectroscopy (FTIR), X-ray diffraction (XRD), and scanning electron microscopy (SEM) were used to analyze the changes in the chemical and physical properties of the OWMECs before and after fungal erosion. These analyses provide technical support for the outdoor application of OWMECs.

## 2. Materials and Methods

### 2.1. Materials

Poplar wood (*Populus canadensis* Moench) with a diameter of approximately 400 mm and a basic density of 0.39 g/cm^3^ was purchased from Langfang Senyuan Wood Co., Ltd., Hebei, China. PF resin was purchased from Dynea Chemical Industry Co., Guangdong, China. The pH, solid content, and viscosity of the PF resin were 10.5 ± 0.2, 46.8% ± 1.0%, and 41.2% ± 2.0%, respectively.

### 2.2. Preparation of Outdoor Wood Mats-Based Engineering Composites (OWMECs)

The wood was manufactured into OWMEC according to a previously published method [2]. Briefly, the wood logs were peeled and split into fiber mats with a thickness of 6 mm. After air-drying, the mats were impregnated with PF resin to achieve a target resin content based on the weight of dried mats. Thereafter, the resin-impregnated wood fiber mats were air dried again to obtain a moisture content of 12 wt.%. Finally, the fiber mats were laminated along the grain in a mold and hot-pressed at 140 °C for 30 min to obtain the OWMEC (400 × 150 × 18 mm^3^). OWMEC samples with different resin contents (8, 13, and 18 wt.%) were obtained with a density of 0.95 g/cm^3^. By changing the mat weight, OWMEC samples with different densities (0.85, 1.00, and 1.15 g/cm^3^) were also obtained with a resin content of 13 wt.%. All the OWMECs were conditioned in a room at 65% relative humidity (RH) and 20 °C for 2 weeks prior to testing.

### 2.3. Decay Resistance

The resistance of the OWMEC specimens and control specimens (poplar wood) against decay by white rot *T. versicolor* fungi and brown rot *G. trabeum* fungi was assessed in accordance with the Chinese Standard GB/T 13942.1-2009 [16]. The dimensions of each specimen were 20 × 20 × 10 mm^3^. Six samples were tested in parallel for each group. White-rot *T. versicolor* fungi or brown-rot *G. trabeum* fungi were inoculated on malt agar medium and pre-incubated for 10 days. Once the mycelium had covered the entire surface of the malt-agar medium, the test specimens were introduced on the sterile glass sticks for 12 weeks at 28 °C and 80% RH. After incubation, the decayed samples were gently cleaned to remove the mycelium adhered to the surface of the samples and oven-dried at 103 °C to constant weight. The percentage mass loss (*ML*) of the samples was calculated after 12 weeks.

(1) $$ML=\;\frac{W_{1}-W_{2}}{W_{1}}\times 100\%$$

where *W*_1_ is the weight of samples before decay and *W*_2_ is the weight of the samples after 12 weeks of incubation.

### 2.4. Chemical Analysis

Changes in the chemical composition of the healthy and decayed OWMEC samples were evaluated following the Chinese standards. The specimens were oven dried, milled, and sieved through a mesh with holes of 0.4 mm. Then, the contents of acid-insoluble lignin (GB/T 2677.8-1994) [17], holocellulose (GB/T 2677.10-1995) [18], and α-cellulose (GB/T 744-1989) [19] were determined.

### 2.5. Fourier Transform Infrared (FTIR) Analysis

FTIR spectra of the OWMECs before and after 12 weeks of fungal exposure were obtained using an FTIR spectrometer (Vertex 70, Bruker, Japan). KBr disks containing 1% of the finely ground samples were employed. Each spectrum was recorded in absorbance units from 1800 to 800 cm^−1^ as an average of 16 scans at a spectral resolution of 4 cm^−1^.

### 2.6. X-ray Diffraction (XRD) Analysis

The crystalline structures of the samples were identified using a Bruker D8 Advance X-ray diffractometer equipped with a Cu Kα X-ray source (*λ* = 1.5404 Å) operated at 40 kV and 40 mA. The X-ray patterns were plotted within the range of 10–80° at a rate of 2° min^−1^. The degree of crystallinity (*Cr*) was determined using the following equation:

(2) $$\mathrm{Cr}=\frac{A_{\mathrm{crystalline}}}{A_{\mathrm{total}}}\times 100\%$$

where *A*_crystalline_ is the sum of all areas of crystallographic reflections and *A*_total_ is the total area of both the crystalline and amorphous contributions.

### 2.7. Scanning Electron Microscopy (SEM) Analysis

Small blocks (3 × 5 × 5 mm^3^) were cut from OWMECs before and after fungal exposure. Sections of 20 µm were sliced off from the cross-section of each block using a sliding microtome until a smooth, clear surface was obtained. Then, all block surfaces were gold-coated and examined using a scanning electron microscope (Hitachi-S4800) at an accelerating voltage of 10 kV.

### 2.8. Statistical Analysis

One-way analysis of variance (ANOVA) was conducted to study the effect of process parameters on decay resistance of the OWEMCs at the 0.05 significance level. Duncan’s tests were employed to multiply compare the properties of OWEMCs with different resin contents and densities.

## 3. Results and Discussion

### 3.1. Mass Loss Analysis

**Figure 1:**
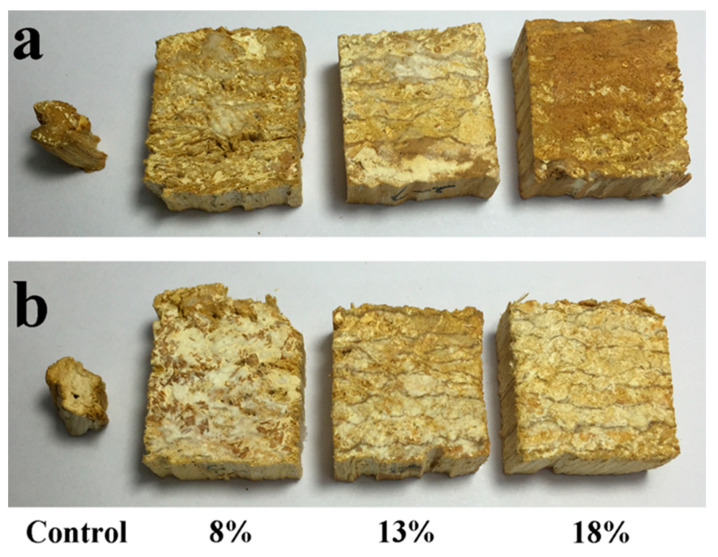
Photographs of wood reference samples (control) and outdoor wood mats-based engineering composite (OWMEC) samples with different resin contents (8%, 13%, and 18%) after 12 weeks of incubation. (**a**) *T. versicolor* decay; (**b**) *G. trabeum* decay.

**Figure 2:**
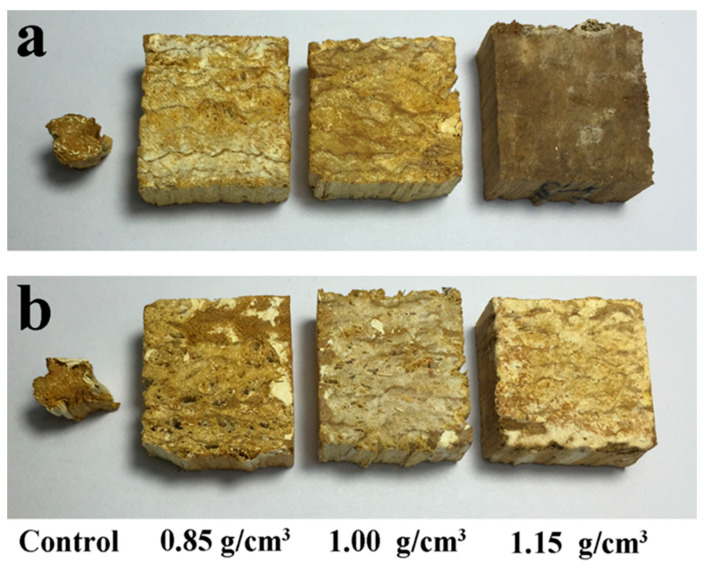
Photographs of wood reference samples (control) and OWMEC samples with different densities (0.85, 1.00, and 1.15 g/cm^3^) after 12 weeks of incubation. (**a**) *T. versicolor* decay; (**b**) *G. trabeum* decay.

The appearance of the specimens exposed to *T. versicolor* and *G. trabeum* fungi is shown in Figure 1 and Figure 2. The original shape of the poplar wood was severely altered, leaving only a small piece of wood. In contrast, OWMEC specimens retained their shape. White decay marks were occasionally observed on the surface of the OWMEC specimens. Furthermore, there were many cracks and holes on the surface of the OWMEC samples with low resin content and density. This indicates that an increase in the resin content and density can improve the decay resistance of OWMEC.

**Figure 3:**
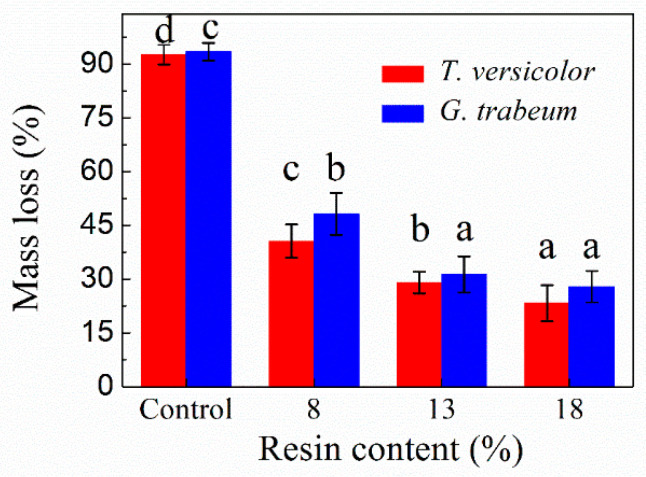
Mass loss of wood reference samples (control) and OWMEC samples with different resin contents after 12 weeks of incubation with *T. versicolor* and *G. trabeum* fungi. For each sample, value bars with the same letter (a, b, c, d) indicate no significant difference at the 0.05 level. Error bars represent the standard deviation.

**Figure 4:**
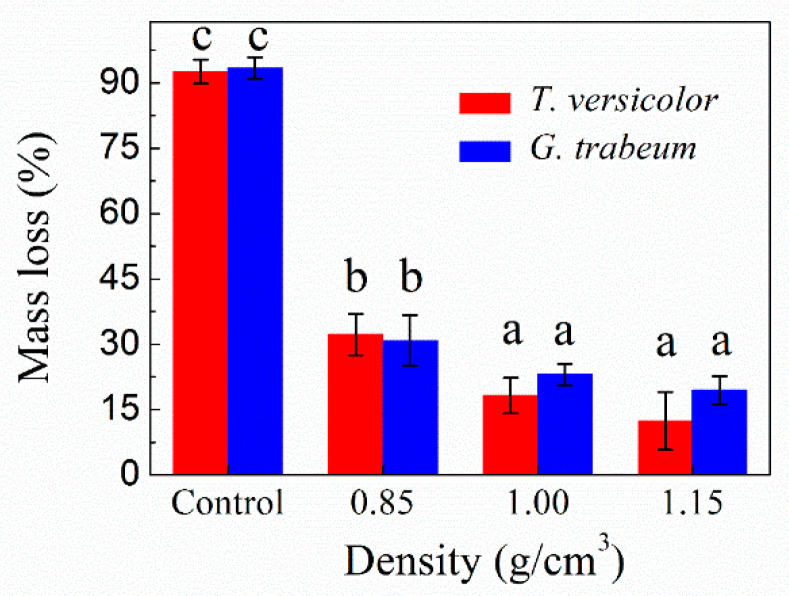
Mass loss of wood reference samples (control) and OWMEC samples with different densities after 12 weeks incubation with *T. versicolor* and *G. trabeum* fungi. For each sample, value bars with the same letter (a, b, c) indicate no significant difference at the 0.05 level. Error bars represent the standard deviation.

Mass loss analysis can predict the potential performance loss of materials. The mass losses of the specimens after 12 weeks of incubation with *T. versicolor* and *G. trabeum* fungi are shown in Figure 3 and Figure 4. For the reference poplar wood samples, mass losses of approximately 92.62% and 93.41% were observed for white- and brown-rot fungi, respectively. This demonstrates that poplar wood is easily destroyed by these fungi. In comparison, the mass loss of the OWMEC samples was significantly reduced, indicating that the fungal resistance is greatly improved when poplar is made into poplar OWMEC. The mass loss of the OWMEC samples was dependent on the resin content and density. As the density and resin content increased, the mass loss decreased. The OWMEC with a resin content of 18% and density of 0.95 g/m^3^ exhibited mass losses as low as 23.24% and 27.88% after fungal attack by *T. versicolor* and *G. trabeum*, respectively. These values increased to 29.05% and 31.27%, respectively, when the resin content and density were 13% and 0.95 g/m^3^, respectively. These results confirm the results of a previous study, which reported that PF resin and densification had a certain inhibitory effect on fungal decay [20]. The OWMEC with a density and resin content of 1.15 g/m^3^ and 13%, respectively, exhibited mass losses of just 12.34% and 19.43% after 12 weeks of incubation with *T. versicolor* and *G. trabeum* fungi, respectively. Therefore, poplar OWMEC with a high resin content and density shows excellent corrosion resistance, making it suitable for use outdoors.

### 3.2. Chemical Analysis

Table 1 and Table 2 show the holocellulose, α-cellulose, and acid-insoluble lignin content of the OWMEC samples before and after fungal decay. The percentage of holocellulose in the decayed OWMEC samples was lower than that in the healthy samples, whereas the percentage of acid-insoluble lignin was increased.

As shown in Table 1, the holocellulose and α-cellulose contents of the OWMEC samples decreased with increasing resin content, whereas the acid-insoluble lignin content increased. This is because the increase in resin content per unit volume and decrease in fiber content led to a decrease in the cellulose content. Regardless of the type of fungi, the holocellulose and α-cellulose contents of the decayed OWMEC decreased as the resin content increased, whereas the acid insoluble lignin increased. With an increase in the resin content, the holocellulose and α-cellulose contents in the *T. versicolor*-exposed OWMEC decreased, whereas the acid-insoluble lignin content increased. The holocellulose and α-cellulose contents in the *G. trabeum*-exposed OWMEC first increased and then decreased, whereas the acid-insoluble lignin gradually increased.

Table 2 shows that the holocellulose and α-cellulose contents of the OWMEC samples increased with increasing density, whereas the acid-insoluble lignin decreased. Regardless of the type of fungi, the holocellulose content of the decayed OWMEC decreased as the density increased, whereas the acid-insoluble lignin content increased.

Microorganisms change the chemical composition of wood during decay. Regardless of the type of fungi, the holocellulose content of the decayed OWMEC decreased as the resin content or density increased, whereas the acid insoluble lignin content increased. Both fungi can simultaneously decompose the major chemical components of wood cell walls [21,22,23]. However, the degradation rate of lignin was not as rapid as that of holocellulose, so the relative content of lignin showed an increasing trend. The amount of holocellulose decomposed by *G. trabeum* fungi was greater than that by *T. versicolor* fungi, indicating that *G. trabeum* has a higher ability to decompose holocellulose than *T. versicolor*. These observations are consistent with previous findings that brown-rot fungi primarily degrade holocellulose [24,25].

### 3.3. FTIR Analysis

**Figure 5:**
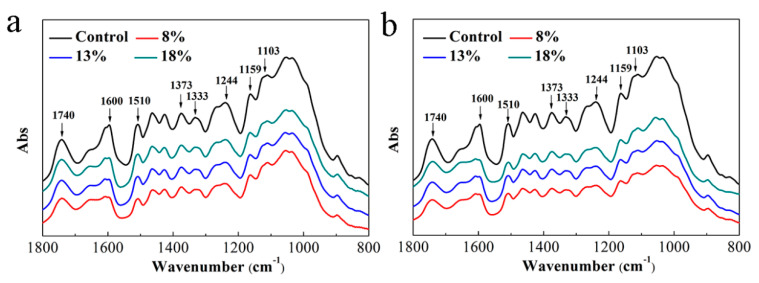
Fourier transform infrared (FTIR) spectra of healthy (control) and decayed OWMEC samples with different resin contents: (**a**) *T. versicolor* decay; (**b**) *G. trabeum* decay.

**Figure 6:**
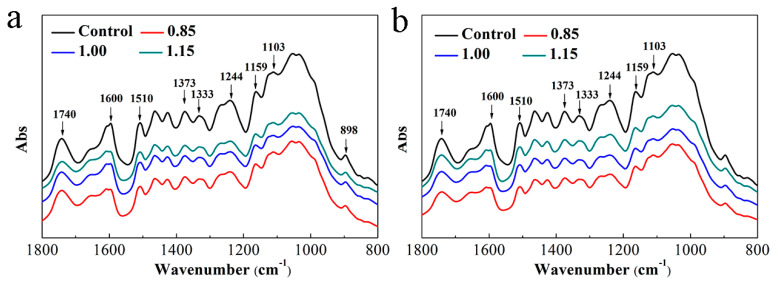
FTIR spectra of healthy (control) and decayed OWMEC samples with different densities: (**a**) *T. versicolor* decay; (**b**) *G. trabeum* decay.

Figure 5 and Figure 6 show FTIR spectra of the OWMECs with different resin contents and densities before and after 12 weeks of fungal attack. The region from 1800 to 800 cm^−1^ is associated with various functional group characteristic of wood components (cellulose, hemicellulose, and lignin). The FTIR spectra of the healthy OWMEC specimen exhibited carbohydrate-associated bands at 1740, 1373, 1159, and 898 cm^−1^ and lignin-associated bands at 1600, 1510, 1462, 1425, 1333, and 1244 cm^−1^ [26,27]. Relative increases and decreases in the intensities of these characteristic absorption peaks indicate chemical changes in the OWMEC. After 12 weeks of decay, the intensity of each peak for each OWMEC sample decreased to some extent. However, the absorption intensity decreased slowly as the resin content or density increased. These results demonstrate that, not only were hemicellulose and lignin decomposed by fungi, but cellulose was degraded to different degrees. The results are similar to those in earlier studies, which showed that both white- and brown-rot fungi degrade carbohydrates and lignin in wood cell walls [28].

In the FTIR spectra of the decayed OWMECs, the intensity of the peak near 1740 cm^−1^, which corresponds to the C=O stretching vibration of the acetyl and carboxyl groups, was reduced. This indicates that the hemicellulose was degraded by both white- and brown-rot fungi. The intensities of the peaks located at 1600 and 1510 cm^−1^, which represent the aromatic skeleton of lignin, decreased significantly, indicating that lignin was decomposed during the fungal decay tests. The intensities of the bands at 1373, 1159, and 898 cm^−1^, which correspond mainly to polysaccharides (hemicellulose and cellulose), decreased after decay treatment. Moreover, the intensities of the peaks at 1333, 1244, and 1103 cm^−1^, which are associated with lignin–carbohydrate complexes, also decreased. The change in the relative intensities of these peaks shows that the consumption of hemicellulose and lignin reached a new balance. The fungal decay-induced chemical changes to the lignin were greater in the OWMEC with a density of 0.85 g/cm^3^ or resin content of 8% than in that with a density of 1.00 g/cm^3^ or resin content of 13%. When the resin content was 18.0% or the density was 1.15 g/cm^3^, the intensity of the aromatic ring structure of lignin in the *T. versicolor* or *G. trabeum*-decayed OWMEC did not change significantly.

### 3.4. XRD Analysis

**Figure 7:**
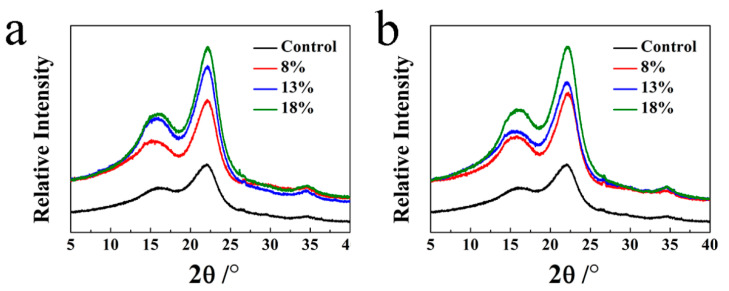
X-ray diffraction (XRD) patterns of healthy (control) and decayed OWMEC samples with different resin contents: (**a**) *T. versicolor* decay; (**b**) *G. trabeum* decay.

**Figure 8:**
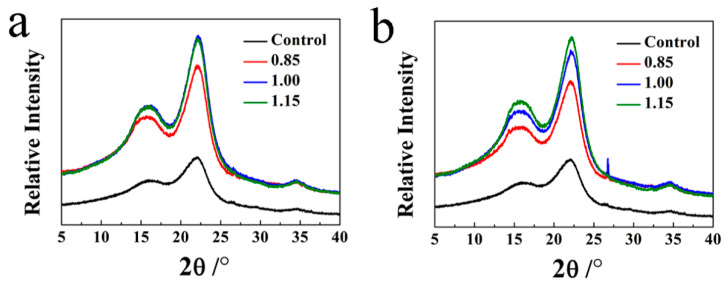
XRD patterns of healthy (control) and decayed OWMEC samples with different densities: (**a**) *T. versicolor* decay; (**b**) *G. trabeum* decay.

The XRD patterns of the OWMECs presented in Figure 7 and Figure 8 show sharp and strong diffraction peaks, indicating the crystalline nature of the OWMEC composites. The peaks at 16.3° and 22.5° were assigned to the (110) and (200) planes, respectively, revealing that the decayed OWMECs with different resin contents and densities possessed a typical wood phase. An increase in the signal of both the (110) and (200) peaks in decayed OWMECs can be observed in Figure 7 and Figure 8. When the XRD intensity is normalized with the (200) peak, the valley at 2*θ* = 18° appears to be slightly lower for the decayed samples than that for the healthy (control) sample. The (200) diffraction peak of decayed OWMECs was narrower than that of the control OWMEC, indicating that the lattice structure of the cellulose crystal zone was destroyed during the decay process.

The *Cr* of the OWMECs increased with increasing resin content and density, regardless of the *T. versicolor* or *G. trabeum* decay treatment (see Table 3 and Table 4). The *Cr* of the healthy (control) OWMEC was 16.34%. The *Cr* increased to 21.81% for the *T. versicolor*-decayed OWMEC with 18% resin content and 21.45% for the *G. trabeum*-decayed OWMEC with 18% resin content, indicating respective increases of 33.48% and 31.27% when compared to the control OWMEC. The *Cr* increased to 23.27% for the *T. versicolor*-decayed OWMEC with a density of 1.15 g/cm^3^ and 23.79% for the *G. trabeum*-decayed OWMEC with a density of 1.15 g/cm^3^, indicating respective increases of 42.41% and 45.59% when compared to the control OWMEC.

The XRD results showed that the *Cr* of the decayed OWMECs increased with increasing resin content and density. The greater *Cr* may be because the fungal degradation rate of hemicellulose is higher than that of cellulose. In contrast to hemicellulose, the decomposition of hydrogen bond-ordered cellulose is a complex procedure. Fackler et al. [29] found that amorphous polysaccharides are more susceptible than crystalline cellulose structures to fungal decay, which results in an increase in overall crystallinity. Furthermore, the higher the density, the higher the fiber content per unit volume.

### 3.5. SEM Analysis

**Figure 9:**
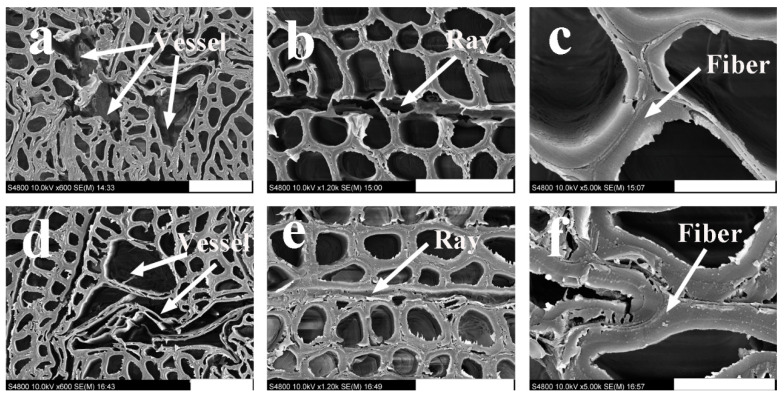
Scanning electron microscope (SEM) images of OWMEC with different resin contents after 12 weeks of incubation with *T. versicolor* fungi. (**a**–**c**) 8% resin content; (**d**–**f**) 18% resin content. Scale bars: (**a**,**d**) 50 μm; (**b**,**e**) 40 μm; (**c**,**f**) 10 μm.

Figure 9 shows the effect of the resin content on the microstructure of *T. versicolor*-decayed OWMEC. Exposure to *T. versicolor* fungi causes ruptures in the walls of all cell elements, such as vessels, fibers, and rays. The cell walls of the vessel of decayed OWMEC with 8% resin content formed rupture gaps and extended to the walls of other cells (Figure 9a). The wood ray was disintegrated, leaving only a small number of residual fragments (Figure 9b). The *T. versicolor* fungi attacked the fiber cells by thinning the cell walls and creating bore holes on the walls (Figure 9c). In contrast, the cell walls of the vessels of the decayed OWMEC with 18% resin content were not seriously damaged, and hyphae were occasionally found in the cells (Figure 9d). The fiber cells away from the vessel walls were found with intact walls, as shown in Figure 9d. A few cracks were observed in the ray cells (Figure 9e), and holes were observed between the fiber cells (Figure 9f). These results demonstrate that the vessels and rays were more vulnerable than the fibers, which were relatively resistant to fungal action. These relative differences are related to deviations in the cell wall thickness, in that the vessel and ray cell walls in poplar are much thinner than the fiber walls.

**Figure 10:**
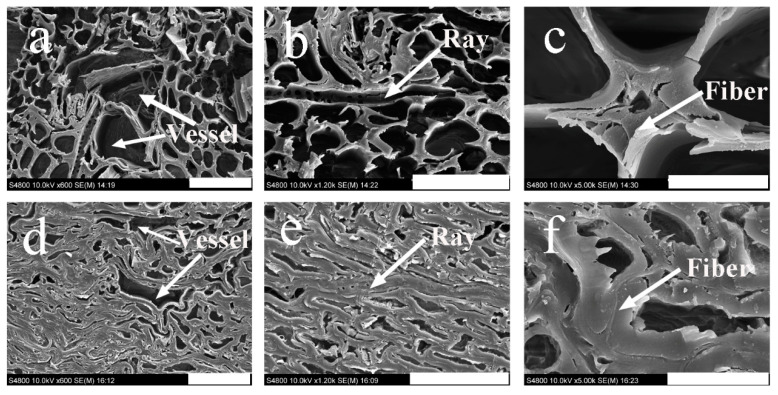
SEM images of OWMEC with different densities after 12 weeks of incubation with *T. versicolor* fungi. (**a**–**c**) 0.85 g/cm^3^; (**d**–**f**) 1.15 g/cm^3^. Scale bars: (**a**,**d**) 50 μm; (**b**,**e**) 40 μm; (**c**,**f**) 10 μm.

Figure 10 shows the effect of density on the microstructure of *T. versicolor*-decayed OWMEC. Colonization of fungal hyphae within the vessel lumina and opening of the vessel walls were observed in the OWMEC with a density of 0.85 g/cm^3^ (Figure 10a). The vessel cell walls contained several discontinuous gaps, indicating degradation by pit erosion. The rays and fibers were also deeply eroded by the *T. versicolor* fungi (Figure 10b,c). Erosion channels with a U-shaped incision appeared on the fiber cell walls, whereby two or more pores had fused together to form large pores on the fiber cell wall (Figure 10b). This illustrates the degradation of the cell walls. Figure 10c reveals that the thickness of the fiber wall decreased from the lumen side to the middle lamellae. Furthermore, erosion troughs formed in the fiber walls. Many large bore holes and loose fiber walls can also be observed in Figure 10c. Notably, in the OWMEC with a density of 1.15 g/cm^3^, all of the cell types retained the compressed cell size and morphology of healthy OWMEC. The morphological changes to the OWMEC with a density of 1.15 g/cm^3^ were not prominent, demonstrating that the extent of damage was greatly reduced.

The SEM images of the decayed OWMEC samples confirmed that the resin content and density were related to the decay resistance. In particular, a high resin content and density enhanced the decay resistance of the OWMEC. Phenolic resins containing various active groups react with the active groups of the cell walls of OWMEC to form stable cross-linking, which effectively improves the corrosion resistance [14,20,30]. The space between the cells was reduced in the higher-density OWMEC, indicating that the diffusion or penetration of hyphae and degrading enzymes was hindered. Moreover, the high density also increased the content of the fiber and phenolic resin, which effectively inhibited fungal corrosion.

## 4. Conclusions

The effects of resin content and density on the resistance of OWMECs to fungal decay were investigated by fungal decay tests. Biological attack resulted in a loss of mass of OWMECs. Depending on the mass loss analysis, a decrease in mass from 32.20% to 12.34% for *T. versicolor* and 30.83% to 19.43% for *G. trabeum* was observed in the OWMECs (density from 0.85 g/cm^3^ to 1.15 g/cm^3^). A decrease in mass from 40.61% to 23.24% for *T. versicolor* and 48.19% to 27.88% for *G. trabeum* was also observed in the OWMECs (resin content from 8% to 18%). The decay resistance of the OWMEC could be enhanced by increasing the resin content or density. The chemical analysis and FTIR measurements showed that brown-rot fungus *G. trabeum* predominantly disintegrated the cellulose and hemicellulose, whereas white-rot fungus *T. versicolor* decayed both holocellulose and lignin; however, the chemical composition was less affected by fungal decal at a higher resin content and density. The *Cr* of the scrimbers increased with increasing resin content and density, regardless of the *T. versicolor* or *G. trabeum* decay treatment. The SEM results confirmed that the resin content and density were related to the decay resistance of OWMEC. In particular, a high resin content and density enhanced the decay resistance of the OWMEC. An appropriate process factor should be performed to improve the outdoor durability of the OWMEC.

## Figures and Tables

**Table 1:** Chemical compositions of OWMEC samples with different resin contents before and after 12 weeks of incubation with *T. versicolor* and *G. trabeum* fungi.

Resin Content (%)	Fungus	Holocellulose (%)	α-Cellulose (%)	Acid Insoluble Lignin (%)
8	-	68.47 (0.10) ^c^	40.58 (0.17) ^b^	25.09 (0.11) ^a^
13	-	64.19 (0.21) ^b^	38.61 (0.54) ^a^	28.12 (0.16) ^b^
18	-	62.64 (0.20) ^a^	37.11 (0.13) ^a^	31.26 (0.16) ^c^
8	*T. versicolor*	63.13 (0.11) ^c^	37.32 (0.08) ^b^	28.86 (0.10) ^a^
13		61.53 (0.16) ^b^	37.31 (0.14) ^b^	33.41 (0.11) ^b^
18		60.46 (0.07^) a^	36.82 (0.10) ^a^	34.44 (0.44) ^c^
8	*G. trabeum*	60.25 (0.13) ^a^	34.98 (0.20) ^a^	31.16 (0.24) ^a^
13		60.39 (0.11) ^a^	36.89 (0.23) ^b^	33.75 (0.08) ^b^
18		60.11 (0.06) ^a^	36.68 (0.10) ^b^	35.48 (0.18) ^c^

Values in parenthesis are standard deviations. For each parameter, average values with different letters (^a^, ^b^, ^c^) in each column indicate a significant difference at the 0.05 level (analysis of variance (ANOVA), followed by Duncan’s multiple range test).

**Table 2:** Chemical compositions of OWMEC samples with different densities before and after 12 weeks of incubation with *T. versicolor* and *G. trabeum* fungi.

Density (g/cm^3^)	Fungus	Holocellulose (%)	α-Cellulose (%)	Acid Insoluble Lignin (%)
0.85	-	63.78 (0.18) ^a^	38.31 (0.13) ^a^	29.63 (0.13) ^c^
1.00	-	64.19 (0.21) ^a^	38.61 (0.17) ^a^	28.12 (0.16) ^b^
1.15	-	65.32 (0.11) ^b^	39.37 (0.08) ^b^	27.31 (0.21) ^a^
0.85	*T. versicolor*	60.29 (0.14) ^a^	35.92 (0.18) ^b^	32.48 (0.18) ^a^
1.00		63.55 (0.08) ^c^	38.99 (0.16) ^c^	32.01 (0.34) ^a^
1.15		62.18 (0.16) ^b^	35.04 (0.11) ^a^	32.64 (0.17) ^a^
0.85	*G. trabeum*	59.32 (0.11) ^a^	35.56 (0.13) ^a^	32.52 (0.16) ^a^
1.00		61.24 (0.10) ^c^	37.77 (0.13) ^b^	32.47 (0.13) ^a^
1.15		60.90 (0.92) ^b^	37.45 (0.06) ^b^	33.18 (0.08) ^b^

Values in parenthesis are standard deviations. For each parameter, average values with different letters (^a^, ^b^, ^c^) in each column indicate a significant difference at the 0.05 level (analysis of variance (ANOVA), followed by Duncan’s multiple range test).

**Table 3:** Crystallinity (*Cr*) of healthy (control) and decayed OWMECs with different resin contents.

Resin Content (%)	Fungus	*Cr* (%)
Control	-	16.34
8.0	*T. versicolor*	16.64
13.0		21.10
18.0		21.81
8.0	*G. trabeum*	18.65
13.0		18.89
18.0		21.45

**Table 4:** Crystallinity (*Cr*) of healthy (control) and decayed OWMECs with different densities.

Density (g/cm^3^)	Fungus	*Cr* (%)
Control	-	16.34
0.85	*T. versicolor*	19.72
1.00		22.81
1.15		23.27
0.85	*G. trabeum*	19.21
1.00		21.78
1.15		23.79

## Data Availability

The data presented in this study are available on request from the corresponding author.
